# A66 EFFECTS OF *HYMENOLEPIS DIMINUTA* EXTRACELLULAR VESICLES ON MOUSE MACROPHAGES AND THEIR MITOCHONDRIA

**DOI:** 10.1093/jcag/gwad061.066

**Published:** 2024-02-14

**Authors:** P Volk, T Allain, N Khan, A Wang, A G Buret, D McKay

**Affiliations:** Physiology and Pharmacology, University of Calgary, Calgary, AB, Canada; Physiology and Pharmacology, University of Calgary, Calgary, AB, Canada; Physiology and Pharmacology, University of Calgary, Calgary, AB, Canada; Physiology and Pharmacology, University of Calgary, Calgary, AB, Canada; Physiology and Pharmacology, University of Calgary, Calgary, AB, Canada; Physiology and Pharmacology, University of Calgary, Calgary, AB, Canada

## Abstract

**Background:**

In recent years, extracellular vesicles (EVs) have emerged as important means of intercellular communication and immune modulation between helminth and host. Earlier work revealed that excretory/secretory products from the rat tapeworm *Hymenolepis diminuta* (HES) have anti-inflammatory properties when applied to macrophages. In this study, we have investigated the immunomodulatory properties of *H. diminuta* EVs (HEVs) on macrophages.

**Aims:**

1. To isolate and characterize bacterial contaminant-free *H. diminuta* EVs (*Hd*-EVs).

2. To determine the effects of *Hd*-EVs on murine macrophage metabolism and LPS-evoked cytokine production.

**Methods:**

Adult *H. diminuta* were cultured for 6h in serum-free RMPI and *Hd-*EVs isolated from the conditioned medium using an exoEasy kit. *Hd-*EV protein content was measured using a micro-BCA kit, and size and quantity were measured using Nanosight tracking analysis. Murine bone marrow-derived macrophages or the human macrophage THP-1 cell line were treated with LPS (10 ng/ml) ± a crude *H. diminuta* extract (HdE), HES, or *Hd*-EVs for 24h. TNF-α levels in the culture medium was measured by ELISA. Macrophage oxidative phosphorylation (OX.PHOS) and glycolysis were measured in real-time using an Agilent Seahorse XF Analyzer.

**Results:**

Analysis of 14 *Hd-*EV samples revealed 4.1 X 10^9^ - 5.48 X 10^10^ particles/mL, with *Hd*-EVs predominantly 100-350 nm in diameter and with a protein content of 64.9-181.6 ng/mL. HdE and HES reduced TNF-α production in LPS-activated macrophages by 39-76% (n=11), while *Hd*-EVs did not lower TNF-α production. Metabolic analysis suggests that HES and *Hd*-EVs can up-regulate macrophage glycolysis, while only *Hd*-EVs exposed macrophages showed increased OX.PHOS (n=6).

**Conclusions:**

We have successfully isolated and begun to characterize EVs from adult *H. diminuta*. Despite the known abilities of HES, we unexpectedly noted that exposure to *Hd*-EVs did not result in a statistically significant decrease in macrophage TNF-α release. However, the increase in OX.PHOS macrophages treated with *Hd*-EVs is something to examine further. The next steps are to determine the contents and immunomodulatory activity of the *Hd*-EVs, thus advancing knowledge of how helminths can manipulate their hosts.

**Funding Agencies:**

NSERC

Clinical Practice

